# Open and Arthroscopic with Mini-Open Surgical Hip Approaches for Treatment of Pigmented Villonodular Synovitis and Concomitant Hip Pathology

**DOI:** 10.1155/2017/3716360

**Published:** 2017-02-23

**Authors:** Bridget Ellsworth, Atul F. Kamath

**Affiliations:** ^1^Perelman School of Medicine, University of Pennsylvania, Philadelphia, PA, USA; ^2^Department of Orthopaedic Surgery, Perelman School of Medicine, University of Pennsylvania, Philadelphia, PA, USA

## Abstract

*Background*. Pigmented villonodular synovitis (PVNS) is a rare benign tumor affecting large joints and prompts excision to prevent local destruction of the joint. The purpose of this case report is to describe two differing surgical approaches for management of PVNS of the hip in patients requiring concomitant treatment for additional hip pathology.* Methods*. This report discusses the presentation, clinical and radiographic findings, and operative management of two contrasting cases of PVNS of the hip. Case 1 describes a 31-year-old female with localized PVNS in addition to a labral tear treated with arthroscopic labral repair followed by tumor excision via a mini-open incision. Case 2 describes a 29-year-old male with more diffuse PVNS in addition to a cam deformity managed with open surgical dislocation of the hip, tumor excision, and restoration of the femoral head/neck junction.* Results*. This report demonstrates two cases of successful excision of PVNS of the hip in addition to addressing concomitant hip pathology in both cases.* Conclusions*. Open surgical dislocation of the hip or arthroscopic surgery with a mini-open incision may be used in appropriately selected patients to successfully excise PVNS lesions in addition to addressing concomitant hip pathology.

## 1. Introduction

Pigmented villonodular synovitis (PVNS) is a rare benign proliferation of synovium involving the joints, bursae, or tendon sheaths [1]. PVNS is generally monoarticular and affects 1.8 patients per million per year; the hip is involved in 15% of cases [2, 3]. Although the origin of PVNS is unknown, it exhibits neoplastic characteristics such as chromosomal anomalies, local tissue invasion, and, rarely, malignant transformation [4]. PVNS may require early surgical excision to limit this local tissue invasion and subsequent joint destruction.

Lesional excision and synovectomy is the treatment of choice for PVNS and can be performed through an open or arthroscopic approach [3]. While the literature on surgical treatment of PVNS of the hip is limited and comprised mainly of case reports, recent studies suggest that arthroscopic techniques can be used safely in carefully selected patients for the excision of PVNS tumors [5–7]. However, if exposure to the lesion is inadequate with arthroscopic techniques or additional hip pathology is present that cannot be addressed using arthroscopy alone, an open technique remains the preferred method of PVNS excision.

The unique aspect of this case report is that both described patients with PVNS lesions required additional treatment for hip pathology beyond tumor excision alone. The purpose of this article is to describe two differing surgical approaches for management of PVNS of the hip in patients requiring concomitant treatment for additional hip pathology. Case 1 describes a patient with PVNS and a labral tear treated with tumor excision through a mini-open incision following arthroscopic labral repair. Case 2 describes a patient with PVNS and a cam deformity managed with open surgical dislocation of the hip, tumor excision, and restoration of the femoral head/neck junction.

## 2. Case Report

### 2.1. Case  1

A 31-year-old woman with a history of right hip developmental dysplasia (DDH) presented with insidious onset of right hip pain, present for several years but significantly worsening over the year prior to presentation. The pain was exacerbated by prolonged sitting and walking and was associated with popping of the hip. Her physical exam was notable for a positive right anterior impingement sign and pain in the right groin exacerbated with log roll, Stinchfield, resisted psoas, and fan kick maneuvers.

**Figure 1:**
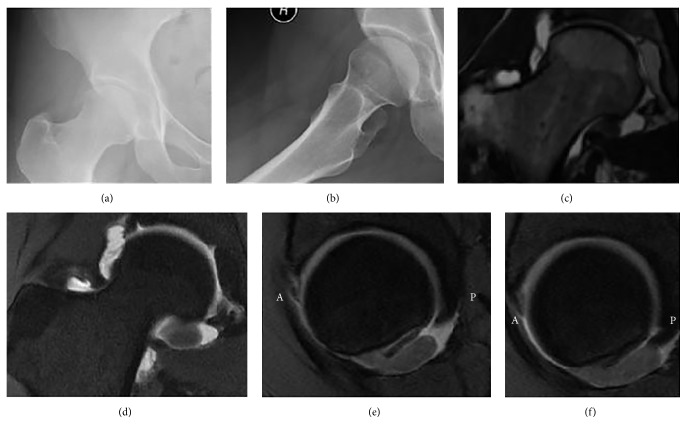
Preoperative anteroposterior (a) and frog-leg radiographs (b) of the right hip in Case 1 demonstrate developmental dysplasia of the hip and the presence of a cross-over sign. Preoperative coronal FIESTA 3D (c), coronal T1-weighted fat-saturated (d), and sagittal proton-density fat-saturated (e, f) magnetic resonance sequences with intra-articular contrast reveal the tumor, a 2.5 cm intermediate to low signal focus within the inferomedial aspect of the right hip joint, in addition to a complex tear of the right anterior and anterosuperior acetabular labrum. A = anterior; P = posterior.

Initial radiographs revealed right hip dysplasia and stigmata of femoroacetabular impingement (FAI), with the right hip exhibiting a cross-over sign (Figures 1(a) and 1(b)). Magnetic resonance (MRI) arthrogram revealed a tumor, 2.5 × 1.3 × 0.8 cm in dimension (anterior-posterior × transverse × craniocaudal) and of intermediate to low signal focus within the inferomedial aspect of the right hip joint. In addition, there was a complex tear of the anterior and anterosuperior acetabular labrum (Figure 1). The tumor raised suspicion for PVNS.

After discussion of the treatment options, the patient elected to undergo right hip arthroscopy with labral repair and possible mini-open incision for mass excision. The patient understood the likelihood of persistent dysplasia requiring osteotomy surgery at a later date after initial management of the tumor.

During the surgery, anterolateral and mid anterior portals were established with the right leg in traction. The labrum was repaired arthroscopically with suture anchor fixation.

The mass within the right hip joint was deemed inaccessible through the arthroscope and rather removed safely and en bloc via a separate mini-open incision along the Smith-Peterson interval. This approach has been described previously for treatment of FAI [8, 9]. An incision was made, starting approximately 2 cm lateral and 1 cm distal to the anterior-superior iliac spine, extending diagonally in line with the muscle belly of the tensor. This incision incorporated the previous mid-anterior portal established during arthroscopy.

**Figure 2:**
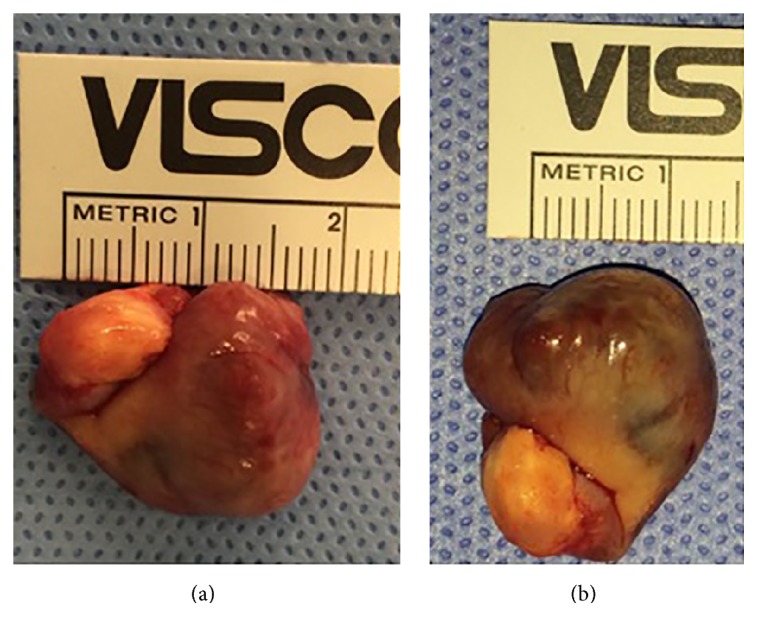
Gross pathology of PVNS from Case 1 (a, b), excised en bloc from the posterior-inferior aspect of the capsule and confirmed to be PVNS on formal pathological analysis.

The mass was located in the posterior-inferior aspect of the capsule. The nodular mass and stalk were excised sharply en bloc (Figure 2) and sent for formal pathologic analysis, which confirmed the presence of PVNS.

The capsule, fascia over the TFL, subcutaneous tissue, and skin were then closed in separate layers along with the portal sites. A sterile dressing was placed over the hip, and a HipRAP was applied for gentle compression. The patient's weight-bearing was protected postoperatively, and a labral repair hip arthroscopy physical therapy protocol was initiated, along with use of a continuous passive motion (CPM) machine.

### 2.2. Case  2

**Figure 3:**
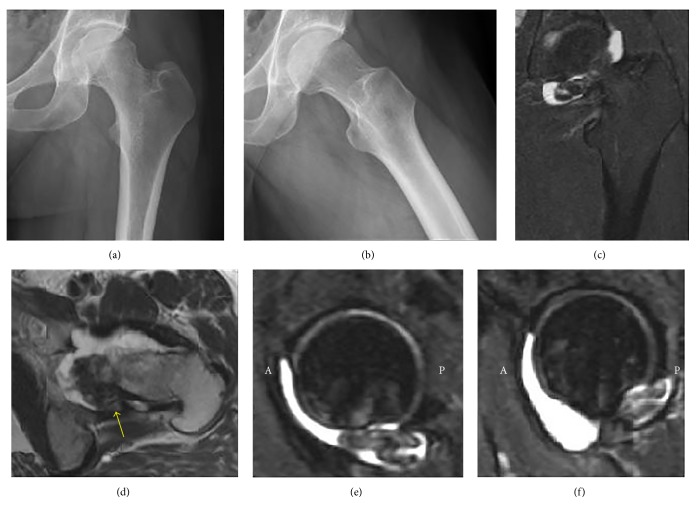
Preoperative anteroposterior (a) and frog-leg radiographs (b) of the left hip in Case 2 demonstrate a cam deformity. Preoperative coronal T2-weighted fat-saturated (c), axial T2-weighted (d), and sagittal T2-weighted fat-saturated (e, f) magnetic resonance sequences with intravenous contrast reveal a multilobulated low signal lesion (arrow in d) measuring approximately 4.0 × 1.1 × 3.4 cm within the posterior/inferior aspect of the hip joint, with an associated large joint effusion. A = anterior; P = posterior.

A 29-year-old male presented with left groin pain that began in adolescence but had been worsening over a two-year period. The pain was exacerbated with activities and was associated with locking and catching, with a history of a number of severe locking episodes in which he was unable to walk for several minutes. Physical exam was notable for pain in the left groin with passive hip rotation, as well as restrictions in range of motion. Outside hospital radiographs were largely unremarkable but did reveal a left-sided cam deformity (Figure 3). A MRI revealed a multilobulated lesion measuring approximately 4.0 × 1.1 × 3.4 cm within the posterior/inferior aspect of the hip joint, with an associated large joint effusion (Figure 3). A computed tomography- (CT-) guided fine-needle aspiration was consistent with PVNS.

The patient elected to undergo a surgical dislocation of the left hip with mass excision and labral/chondral treatment, as an arthroscopic approach or anterior mini-open incision would likely not provide adequate circumferential hip access for excision of the large mass tethering the inferior femoral neck.

**Figure 4:**
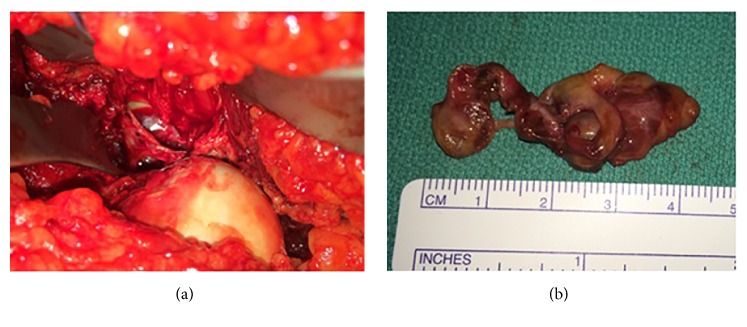
Intraoperative image of a surgical dislocation of the left hip in Case 2, which allowed for adequate exposure to visualize and excise the mass (a). Gross pathology of the tumor from the same case is depicted (b), excised en bloc from the posterior-inferior aspect of the capsule and confirmed to be PVNS on formal pathological analysis.

With the patient under general anesthesia, a surgical dislocation of the left hip was performed using the technique described by Ganz et al. [10] (Figure 4). The synovial mass was excised en bloc down to the stalk (Figure 4). The stalk was also excised and cauterized.

In addition to removal of the mass, the femoral head/neck junction was reshaped due to the presence of a cam deformity. Using a combination of osteotomes and a burr, the gentle waist of the femoral head and neck junction was reconstituted. Offset templates were used to verify extent of decompression needed. The retinacular vessels were identified and protected at all times, and gentle continuous irrigation was performed to exposed cartilage surfaces of the acetabulum and femur throughout. Bone wax was applied to the exposed bony surfaces after offset correction. The hip was reduced, and a very nice labral seal could be seen through the peripheral compartment.

The labrum was well reduced and stable onto the bony rim. The capsulotomy was closed without undue tension and with partial thickness (nonarticular sided) bites in interrupted fashion. The trochanteric flip osteotomy was reduced and fixed with screws. The trochanteric bursa was closed over the screws, and the subvastus approach to the femur was closed. The patient's weight-bearing was protected immediately postoperatively, and a surgical hip dislocation postoperative protocol was followed.

## 3. Discussion

The authors report two contrasting cases of PVNS of the hip in addition to concomitant hip pathology treated successfully using different approaches. While both lesions were located in the posterior-inferior aspect of the capsule, the patient's tumor in Case 1 was smaller and easily accessible via a mini-open approach following labral repair via hip arthroscopy, obviating the need for a larger incision. The patient's mass in Case 2 was larger and would have been difficult to visualize and excise using a minimally invasive approach. Furthermore, treatment of the large cam deformity could be performed concurrently through the open dislocation approach.

While PVNS is a benign lesion, it can be locally invasive and destructive and may require complete removal to minimize risk of recurrence. Thus, the patient in Case 2 elected for a more extensive procedure with surgical dislocation of the hip in order to ensure complete excision of the tumor. Additionally, both patients had concomitant hip pathology that required attention and treatment at the time of surgery.

This report demonstrates that PVNS of the hip must be managed on a case-by-case basis, as multiple factors, such as size and location of the lesion as well as additional hip pathology, can influence surgical management. A recent report by Shoji et al. describes two young patients (<30 years old) with advanced PVNS of the hip, both with evidence of radiographic joint-space narrowing at the time of presentation [11]. Instead of performing a combined synovectomy and arthroplasty, the authors opted for a joint-preserving procedure in these young patients. Both patients had areas of cartilage loss over weight-bearing aspects of the femoral head. In each patient they performed a synovectomy, a transtrochanteric rotational osteotomy (TRO) to transfer an area of femoral head with remaining cartilage to a weight-bearing area, and microfracture of areas with residual osteochondral lesions. In one patient autologous osteochondral transplantation (AOT) was also used for three areas of full-thickness cartilage defect. Both patients had similar good outcomes postoperatively at 2- and 2.5-year follow-up with no PVNS recurrence and minimal symptoms.

Both arthroscopic and open procedures have been successful in preventing recurrence of PVNS of the hip [3, 5]. However, Vastel et al. described a high rate of secondary osteoarthritis in patients treated for PVNS using an open approach with surgical dislocation of the hip [3]. This may be due to joint destruction already present at the time of PVNS diagnosis and unrelated to the surgical approach used. However, given this high rate of secondary osteoarthritis, there has been a recent push for minimally invasive approaches in the appropriate patient for treatment of PVNS. Byrd argued that while arthroscopy may not prevent the development of osteoarthritis depending on the damage already present at the time of surgery, it may prevent the need for two invasive procedures assuming the eventual need for arthroplasty [12].

However, due to limitations in exposure during arthroscopy, surgical dislocation of the hip is still required to excise certain lesions, especially large lesions in the posterior-inferior capsule, as in Case 2. As stated previously, many cases of PVNS already have significant secondary joint damage by the time of diagnosis and treatment. A recent systematic review by Levy et al. found a high revision rate in patients undergoing surgery for PVNS (about 1 in 4 patients) [13]. While this rate was similar for patients who had undergone synovectomy alone compared to a combined synovectomy and arthroplasty, the time to revision surgery was significantly shorter in those patients who had undergone synovectomy alone. Della Valle et al. reported follow-up data ranging from 2 to 23 years on 7 patients with PVNS [14]. They found a high rate of revision surgery in patients who had combined synovectomy and arthroplasty. Of 4 patients who underwent total hip arthroplasty (THA), 2 required revision arthroplasty due to aseptic loosening. However, none of the 4 patients with combined synovectomy and arthroplasty had a recurrence of PVNS. The one patient treated with synovectomy alone had recurrence of PVNS 9 years after synovectomy and underwent a repeat synovectomy and THA at that time. The patient in Case 2 elected to undergo mass excision with synovectomy alone while simultaneously addressing intra-articular pathology (cam lesion).

This case report demonstrates that patients with PVNS tumors and concomitant hip pathology may be successfully treated using multiple techniques. There are myriad factors that determine which technique should be used, but location and size of the lesion, additional hip pathology, surgeon experience and expertise, and patient preference should all play a role in the decision-making process. Further research is necessary to determine whether surgical approach to PVNS affects long-term outcomes and progression to secondary osteoarthritis.

## 4. Conclusion

Excision of PVNS of the hip is necessary to halt progression and local destruction of the joint. Patients with PVNS lesions of the hip may have additional hip pathology that must be addressed at the time of surgery. Open surgical dislocation of the hip or arthroscopic surgery with a mini-open incision may be used in appropriately selected patients to successfully excise PVNS lesions in addition to addressing concomitant hip pathology.
